# Cesium and strontium tolerant *Arthrobacter* sp. strain KMSZP6 isolated from a pristine uranium ore deposit

**DOI:** 10.1186/s13568-016-0247-3

**Published:** 2016-09-13

**Authors:** Pynskhem Bok Swer, Santa Ram Joshi, Celin Acharya

**Affiliations:** 1 Department of Biotechnology and Bioinformatics, North Eastern Hill University, Umshing, Mawlai, Shillong, 793022 India; 2 Molecular Biology Division, Bhabha Atomic Research Centre, Trombay, Mumbai, 400085 India; 3 Homi Bhabha National Institute, Anushakti Nagar, Mumbai, 400094 India

**Keywords:** Cesium, Strontium, Tolerance, Binding

## Abstract

*Arthrobacter* sp. KMSZP6 isolated from a pristine uranium ore deposit at Domiasiat located in North-East India exhibited noteworthy tolerance for cesium (Cs) and strontium (Sr). The strain displayed a high minimum inhibitory concentration (MIC) of 400 mM for CsCl and for SrCl_2_. Flow cytometric analysis employing membrane integrity indicators like propidium iodide (PI) and thiazole orange (TO) indicated a greater sensitivity of *Arthrobacter* cells to cesium than to strontium. On being challenged with 75 mM of Cs, the cells sequestered 9612 mg Cs g^−1^ dry weight of cells in 12 h. On being challenged with 75 mM of Sr, the cells sequestered 9989 mg Sr g^−1^ dry weight of cells in 18 h. Heat killed cells exhibited limited Cs and Sr binding as compared to live cells highlighting the importance of cell viability for optimal binding. The association of the metals with *Arthrobacter* sp. KMSZP6 was further substantiated by Field Emission-Scanning Electron Microscopy (FE-SEM) coupled with Energy dispersive X-ray (EDX) spectroscopy. This organism tolerated up to 1 kGy ^60^Co-gamma rays without loss of survival. The present report highlights the superior tolerance and binding capacity of the KMSZP6 strain for cesium and strontium over other earlier reported strains and reveals its potential for bioremediation of nuclear waste.

**Electronic supplementary material:**

The online version of this article (doi:10.1186/s13568-016-0247-3) contains supplementary material, which is available to authorized users.

## Introduction

Radionuclides released from nuclear testing, weapon production, nuclear fuel processing, accidental spills and emissions have resulted in contaminated soil, water and sediments. Cesium (^137^Cs) and strontium (^90^Sr) are natural by-products of nuclear fission or released by catastrophic accidents like the nuclear meltdown and explosion of Chernobyl or Fukushima nuclear power plant (Ginzburg and Reis 1991; Kinoshita et al. 2011). Owing to physicochemical resemblance to potassium and calcium respectively, cesium and strontium may enter the food chain via K^+^ and Ca^2+^ transport pathways (White et al. 2002; Qi et al. 2008). These radionuclides pose serious threat to biota for their persistent presence following a pulse of contamination due to their long half lives (30.17 and 28.8 years for ^137^Cs and ^90^Sr respectively) (Yasunari et al. 2011). The beta and gamma radiation emitted by these radionuclides are associated with the elevated risk of cancer and therefore present high radiological concern (Hatch et al. 2005). In addition to radiological hazard, the chemical toxicity of Cs^+^ and Sr^2+^ is well documented (Bonatti et al. 1983; Morohashi et al. 1994; Ozgur et al. 1996). While cesium has been reported to cause fatigue, muscle weakness, palpitations and arrhythmia, strontium impairs mineralization of the bone which is attributed to osteomalacia and rickets (Cabrera et al. 1999; Melnikov and Zanoni 2013).

Cesium and strontium released in the environment may occur as elemental, oxide, precipitates, ionic, inorganic and organic-complexes (Francis 1985). Several microbes have been isolated from such contaminated environment and used in the context of Cs^+^ and Sr^2+^ bioremediation (Francis 1985; Tomioka et al. 1992; Kuwahara et al. 2011; Dekker et al. 2014). Cs^+^ is the most toxic metal among alkali metals for the microorganisms. The toxicity results from either reduced influx or increased efflux of K^+^ (Bossemeyer et al. 1989; Jung et al. 2001). Cesium binding is thought to be facilitated by potassium transport system(s) in various microorganisms (Borst-Pauwels 1981; Bossemeyer et al. 1989; Avery 1995). Similarly, the binding of Sr^2+^ is mediated in yeast by Ca^2+^ transport and distribution system (Avery and Tobin 1992; Heuck et al. 2010). Sr^2+^ substituted for Ca^2+^ without imparting any major cellular toxicity in cellular processes like spore formation, flagellum biosynthesis or enzyme inactivation in various microorganisms (Robinson et al. 1992; Beveridge and Murray 1976; Goodwin et al. 1996).

Microorganisms in terrestrial subsurface environments are known to play a significant role in the biogeochemical cycling of elements including radionuclides by altering their speciation, solubility and sorption properties. Native bacterial community assemblages from the subsurface soils of pristine uranium (U)- rich deposit of Domiasiat, Meghalaya located in North-Eastern part of India exhibited features like (a) significant tolerance to U (VI) and heavy metals like Cu (II), Pb (II), Cd (II) (b) viability under acidic conditions (pH 3.5) and the (c) presence of phosphatases and P_IB_ type ATPases (Kumar et al. 2011, 2012, 2013a, b; Nongkhlaw et al. 2012). The mobility of uranium in the environment depends on its speciation and redox state as well. Uranium is present as U (VI) under oxidizing conditions. Under reducing conditions, uranium predominates as insoluble and immobile U (IV) as mineral uraninite, UO_2_[s]. The uranium- rich deposit in Domiasiat is represented by acidic soil augmented with elevated concentrations of U (VI) and heavy metals like Cu (II), Pb (II), Cd (II) (Table 1) (Kumar et al. 2012). Microorganisms colonizing such extreme environment are strongly affected by the selection pressure resulting from pH, oxidative stress and high metal contents. These organisms are likely to harbor myriad of strategies for environmental acclimation and can serve as useful models for investigating the metal transport and toxicity at the cellular and molecular level. Several strains of *Arthrobacter* were isolated previously from the aforementioned site (Kumar et al. 2012). *Arthrobacter* strains are ubiquitous and have been found in the common soils and extreme environments such as deep subsurface, arctic ice, sediments from a high-level nuclear waste plume, uranium mine site, toxic and the heavy metal contaminated sites (van Waasbergen et al. 2000; Fredrickson et al. 2004; Hanbo et al. 2004; Macur et al. 2004; Suzuki and Banfield 2004).

**Table 1:** Physicochemical properties of the soil sample from KMS1 site (adapted from Kumar et al. 2012)

GPS	Altitude (m)	pH	OC (g kg^−1^)	Elements (mg kg^−1^)					
				U	Cu	Pb	Cd	Cs^a^	Sr^a^
N25^o^19.285 E91^o^12.594	824	5.6	7.5–8	480	28.8	23	0.9	0.1	0.45

OC, Organic carbon

^a^ Represents the values determined in this study

*Arthrobacter* sp. strain KMSZP6 (hereafter referred to as KMSZP6 strain) isolated from Domiasiat site was found to tolerate 4 mM U (VI), 4 mM Cu (II), 1 mM Cd (II) and 1 mM Pb (II) (Kumar et al. 2012, 2013a). The present investigation was undertaken to evaluate the tolerance of KMSZP6 strain for cesium and strontium. Data obtained using spot assays, flow cytometry, energy dispersive X-ray Fluorescence (EDXRF) analyses and high resolution field emission scanning electron microscopy (FE-SEM) based imaging coupled with energy dispersive X-ray (EDX) spectroscopy established the notable tolerance and binding capabilities of this strain for Cs^+^ and Sr^2+^.

## Material and Methods

### Sample site and molecular characterization of KMSZP6 strain

Soil samples from the proposed mine site (Kylleng mine site, KMS1, N25°19.285′ E91°12.594′) of uranium ore deposit (Additional file 1: Fig. S1) were collected in triplicate in separate sterile plastic bags and transported on ice to the laboratory. Physicochemical analyses were conducted on this collection of the soil samples as described earlier (Kumar et al. 2012). Cs^+^ and Sr^2+^ concentrations were determined by digestion of a subsample of the soil (1 g) with 25 mL HNO_3_ and 2 mL H_2_O_2_, followed by filtration through Whatman filter paper (No.12) and analysis using an inductively coupled plasma mass spectrometer (ICP-MS) (Perkin Elmer Elan DRC, USA).

A pure culture of *Arthrobacter* sp. strain KMSZP6 was previously isolated from the soil sample as described earlier (Kumar et al. 2012). 16S rRNA gene sequences of the representative strains of *Arthrobacter* were retrieved from NCBI with their corresponding accession numbers including the type strain *Arthrobacter nicotinovorans* (X80743.1) and were aligned with that of *Arthrobacter* sp. strain KMSZP6 using MEGA 5.1. The basic local alignment search tool (BLASTX) was used to determine the phylogenetic neighbors of the 16S rRNA genes from the GenBank database (National Center for Biotechnology Information, Bethesda, USA) (Altschul et al. 1997). Phylogenetic analysis was performed by MEGA 5.1 using an average of 1400 nucleotides of 16S rRNA encoding DNA sequences (Tamura et al. 2011). The 16S rRNA gene sequence of *Deinococcus radiodurans* (AY940039.1) was taken as an outgroup. Neighbor joining method was used to construct the phylogenetic tree with 1000 bootstrap values. The partial 16S rRNA gene sequence of this strain was submitted to the NCBI GenBank under the accession number JF768707.

### Sensitivity analysis of KMSZP6 strain

KMSZP6 cells were grown to the exponential phase in low phosphate medium (LPM) (Poole et al. 1989) which contained (in grams/litre): Tris, 14.5; NaCl, 4.68; KCl, 1.5; NH_4_Cl, 1.0; glycerol, 5; Na_2_SO_4_, 0.043; CaCl_2_, 0.03, pH 7.5. Volumes of 10 µL from a dilution series of an exponential phase cell culture (equivalent to OD_600 nm_ 1) were spotted on LPM agar plates amended with 0–400 mM of CsCl (Hi Media, India) or SrCl_2_ (Hi Media, India). Cells exposed to 0–400 mM NaCl (Hi Media, India) under similar experimental conditions were included as control to discriminate between osmotic stress induced toxicity (resulting from high ionic strength of the metal solutions) and metal induced toxicity. LPM is well suited for metal sensitivity studies because it contains negligible phosphate and therefore lessens the possibility of metal precipitating as metal phosphate (Kumar et al. 2013b). The plates were incubated for 3 days at 37 °C. The lowest concentration of the metal that completely inhibited the growth of the test organism was termed as the minimal inhibitory concentration (MIC) (Yilmaz 2003).

### Cs^+^ and Sr^2+^ exposure, Fluorescence probe staining and Flow cytometric analysis

Exponential phase cells were harvested by centrifugation, washed with dH_2_O and exposed to 0–400 mM of NaCl or CsCl or SrCl_2_ in Low Phosphate medium (LPM) and incubated for 24 h at 37 °C with shaking (220 rpm). Cells were harvested by centrifugation and washed with dH_2_O. The cell pellets were subsequently resuspended in staining buffers (Phosphate-buffered saline PBS, 1 mM EDTA, 0.01 % Tween 20, 0.1 % sodium azide, pH 7.4). Propidium Iodide (PI) and Thiazole Orange (TO) (BD™ Cell viability kit, BD Biosciences USA) were used for the determination of live/dead cells. Control and metal treated cells (equivalent to OD_600 nm_ 1) were stained with propidium iodide (PI) and thiazole orange (TO) as per the manufacturer’s instructions. Each dye solution, TO and PI was added to the cell suspension to a final concentration of 420 nM and 48 µM respectively and incubated for 30 min at room temperature before proceeding to flow cytometric analysis. The stained cells were analyzed by FACSCalibur™ flow cytometer (BD Biosciences, USA) equipped with a 15 mW, 488 nm argon-ion laser; fluorescence was collected by three coloured photomultiplier tubes with fluorescence emission filters (FL1 530/30 nm, FL2 585/42 nm and FL3 650 LP). Lower and upper fluorescence limits that included most of the cells in the live control (more than 99.9 %) were determined. Within those limits, stained cells were considered as the intact cells. Stained cells with fluorescence greater than the upper limit were considered permeabilized cells. A Forward-scattered light (FSC) vs Side-scattered light (SSC) plot was set up around the bacterial population followed by another region of stained bacterial population in the FL2 vs SSC dot plot. FL3 vs FL1 dot plot was obtained based on the gated combinations of FSC, SSC and FL2 (BD Biosciences, USA). After completing the set up, 10,000 cells were analyzed per sample. Live/injured/dead cell populations were analyzed with Cell-Quest Pro software from Becton–Dickinson (BD Biosciences, USA).

### Irradiation studies

For irradiation studies, exponential phase KMSZP6 cells (OD_600 nm_ 1) were exposed to gamma radiation from ^60^Co source at a dose rate of 35.7 Gy/min (Gamma Chamber GC-5000, Bhabha Atomic Research Centre, Mumbai, India). Irradiated cells were washed twice with LPM medium. Volumes of 10 µL from a dilution series of the irradiated cell culture were spotted on LPM agar plates and incubated under usual growth conditions. Radiation tolerance of KMSZP6 was assessed in terms of post-irradiation survival.

### Cs^+^ and Sr^2+^ binding assays

Cells were harvested in the exponential phase of growth by centrifugation (7000 rpm for 3 min), washed with distilled water and used for Cs^+^ and Sr^2+^ binding studies. Cells washed with isotonic saline (8.5 g NaCl l^−1^) and analyzed independently for Cs^+^ and Sr^2+^ binding studies yielded results similar to those cells washed with distilled water. Sterile experimental (LPM) solutions (50 ml) were supplemented with either 75 mM CsCl or SrCl_2_ and were allowed to equilibrate for 30 min under aerobic conditions. Experiments were initiated by inoculating *Arthrobacter* sp. KMSZP6 cells (equivalent to OD_600 nm_ 1) in the medium and incubating them under continuous shaking at pH 7.5 for 24 h at 37 °C. The cell density did not change measurably during such incubation. Aliquots were withdrawn at regular time intervals and centrifuged at 10,000 rpm for 3 min. Estimation of soluble fraction of Cs^+^ in culture supernatants was done using the method proposed by Huey and Hargis (1967). The supernatants were acidified with perchloric acid (6 N) and diluted with distilled water. For Cs^+^ precipitation, 12-molybdophosphoric acid solution (14 % w/v) was added to the acidified supernatants and the resulting mixtures were allowed to stand for 15 min. The precipitates (containing cesium) were centrifuged, washed with 1.2 N perchloric acid solution and were dissolved completely in borate buffer (pH 9). The absorbance was recorded spectrophotometrically at 226 nm. Similarly, soluble Sr^2+^ was analyzed using the method adapted from Dunstone and Payne (1959). EDTA (0.005 M) and buffer solution containing 1.2 % (wt/vol) of ammonium chloride and 11.4 % (v/v) of ammonia solution (sp. gr. 0.880), pH 10 were added to the culture supernatants. The resulting mixtures were further diluted with distilled water and the absorbance was recorded spectrophotometrically at 225 nm. Heat killed or non-viable cells, obtained by incubation of the cells in boiling water for 15 min, were also included for Cs and Sr binding assays. The non- viability of the heat killed cells was ascertained by flow cytometry and their cellular integrity was assessed by microscopy.

KMSZP6 cells challenged with 75 mM of CsCl or SrCl_2_ for 24 h were examined by Energy dispersive X-ray fluorescence (EDXRF) spectrometer. The metal exposed cells were washed with distilled water to remove loosely bound surface Cs^+^ or Sr^2+^, dried at 60 °C, ground to homogeneity and pressed into pellets. The pellets were glued to a mylar film on a sample holder and placed directly in the X-ray beam for Cs^+^ and Sr^2+^ determination using radioisotope source of 100 mCi^241^Am as described earlier (Acharya et al. 2009).

### Field emission-scanning electron microscopy (FE-SEM)—energy dispersive X-ray (EDX) analyses

Cells challenged with 75 mM of Cs^+^ or Sr^2+^ for 24 h were subjected to field emission scanning electron microscopy and compared with the control, metal unchallenged cells. Control and metal (Cs or Sr) treated cells were washed with dH_2_O and fixed using 2.5 % of glutaraldehyde (Sigma, USA) for 1 h at room temperature. Fixed cells were washed twice with dH_2_O and were subsequently dehydrated in an alcohol series (10, 30, 50, 70 and 100 % ethanol) for 5 min in each alcohol solution and left to dry in a desiccator overnight. The specimens were mounted onto the sample holder with carbon-conductive adhesive tapes and coated with gold using a sputter coater. Samples were viewed using a field-emission scanning electron microscope (FE-SEM) (Carl Zeiss Auriga, Germany). Elemental composition of Cs or Sr loaded cells was determined by EDS (Oxford Instruments with Silicon Drift Detector, SDD- X-Max^N^) attached to FE-SEM.

Cells treated with NaCl (400 mM for 24 h) were also subjected to FE-SEM analyses to examine the morphological changes under increased osmotic stress resulting from high ionic strength of salt solution (400 mM Na^+^).

### Statistical analyses

Each treatment included three replicates. Each experiment was repeated at least three times. The observed variation was less than 10 %. Representative data from one experiment (means with standard error values) are shown.

## Results

### Isolation of KMSZP6 strain

**Fig. 1:**
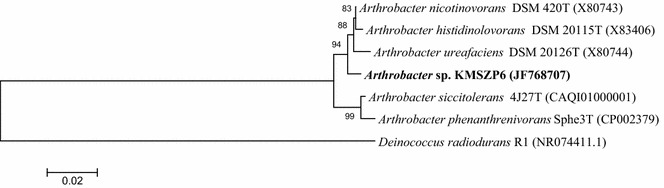
Evolutionary distance dendrogram of *Arthrobacter* sp. KMSZP6 strain with sequences of the representative *Arthrobacter* strains from NCBI database (based on >1400 nucleotides of 16S rRNA). *Deinococcus radiodurans (*AY940039.1) was taken as the outgroup organism. The *scale bar* corresponds to the expected number of changes per nucleotide position

KMSZP6 strain was isolated previously from the soil sample at KMS1 site of U ore deposit (Kumar et al. 2012). Table 1 represents the physicochemical characteristics of the soil sample. The soil contained moderate concentration of organic carbon, high concentration of uranium (480 mg kg^−1^) and low concentrations of cesium and strontium (0.1 and 0.45 mg kg^−1^ respectively). The pH of the soil sample was 5.6. Other metal contents were found to be within the range of the background concentration of trace elements consistent with non-anthropogenic soils (Burt et al. 2003). The identity of *Arthrobacter* sp. KMSZP6 closely matched that of *Arthrobacter nicotinovorans* owing to its high sequence similarity and phylogenetic relatedness to the closest type strain (sequence X80743) (Fig. 1). The strain was deposited with National Collection of Industrial Microorganisms (NCIM) (www.ncl-india.org/files/NCIM), Pune, India under the accession number 5597.

### Cs^+^, Sr^2+^ and radiation tolerance

**Fig. 2:**
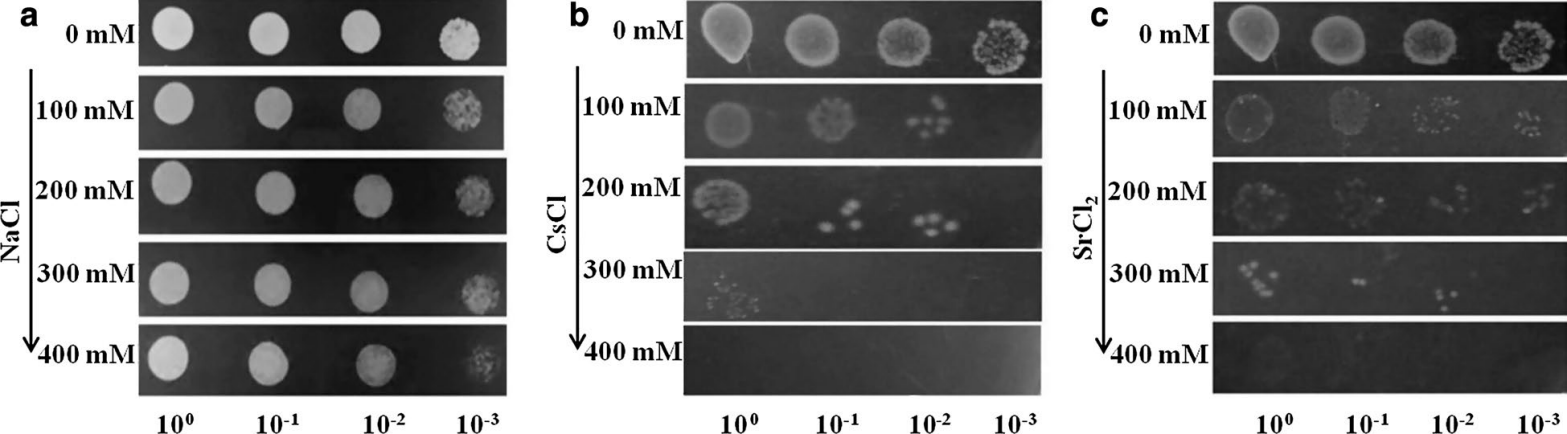
Elucidation of minimum inhibitory concentration (MIC) of KMSZP6 strain for Cs^+^ and Sr^2+^. Starting with exponential phase cell suspensions equivalent to OD_600 nm_ 1, 10 μL from a dilution series was spotted onto LPM agar plates supplemented with concentrations varying from 0 to 400 mM of **a** NaCl or **b** CsCl or **c** SrCl_2_. The plates were incubated for 3 days at 37 °C. The minimum concentration (MIC) which completely inhibited visible growth of the test organism was obtained at 400 mM for CsCl or SrCl_2_

Spot assays revealed the growth sensitivity of KMSZP6 strain at 100 mM and its complete inhibition (MIC) at 400 mM of CsCl or SrCl_2_ (Fig. 2b, c) in contrast to NaCl treatment (Fig. 2a) where no such sensitivity was observed even up to 400 mM of NaCl. The cells were found to tolerate as much as 300 mM of Cs or Sr although the isolation site (KMS1) harboured modest concentrations of these metals (0.75 µM and 5.13 µM of Cs and Sr, respectively) (Table 1).

Following 24 h exposure to 0–400 mM of Cs^+^ or Sr^2+^, flow cytometric analysis using live/dead staining dyes like propidium iodide (PI) and thiazole orange (TO) was applied to discriminate live, dead and injured KMSZP6 cells. The cells (a) with active metabolism and no leaking membrane were designated as the viable (live) cells, (b) those with damaged cell membranes through which both stains (PI and TO) diffused to different degrees were termed as injured cells and (c) those without a cell membrane or a damaged one which allowed PI to easily diffuse were termed as dead cells. The injured cells could be active but not enough to be detected as live cells or could be dying cells with intact membranes (Joux and Lebaron 2000).

**Fig. 3:**
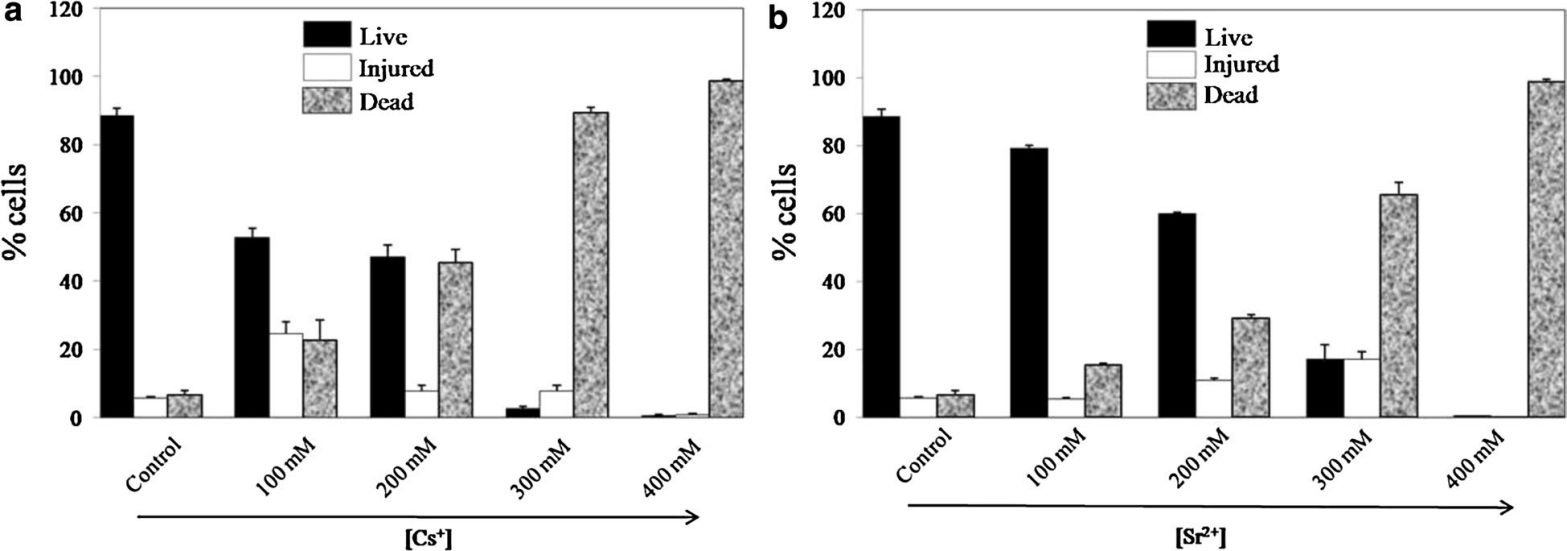
Flow cytometric analyses of Cs^+^ and Sr^2+^ toxicity. Exponential phase cells (equivalent to OD_600 nm_ 1) were exposed to 0-400 mM of Cs (**a**) or Sr (**b**) for 24 h, stained with propidium Iodide (PI) and thiazole orange (TO) and were gated on combined parameters of FSC, SSC and FL2 (see text for details). Data points represent percentage subpopulation of live, injured and killed cells with varying concentrations of Cs^+^ and Sr^2+^ with corresponding standard error values

Flow cytometric analysis using the membrane integrity indicators demonstrated a significant reduction in the viable cell populations following exposure to the increasing concentrations of Cs^+^ or Sr^2+^ suggesting a dose dependent relationship (Fig. 3a, b). The loss of membrane integrity represented by the dead cell populations was more abrupt and extensive with Cs^+^ than with Sr^2+^ (Additional file 2: Fig. S2). While ~78.9 % of the cells were viable following 100 mM Sr^2+^ exposure (Fig. 3b), only 53.2 % of the cells were found to be intact and viable after equivalent Cs^+^ exposure (Fig. 3a) indicating higher sensitivity of the cells towards Cs^+^. Complete loss of the live cell populations at 400 mM of Cs^+^ or Sr^2+^ (Fig. 3a, b; Additional file 2: Fig. S2) indicating the loss of culturability was consistent with the observed MIC value of 400 mM in the spot assays (Fig. 2b, c). In contrast, viability of the cells was largely unaltered following exposure to 400 mM Na^+^ (data not included) complimenting the results obtained from spot assays (Fig. 2a).

Based on post-irradiation survival or viability, KMSZP6 cells displayed a noteworthy radiation tolerance up to 1 kGy (Additional file 3: Fig. S3).

### Cesium and strontium binding

**Fig. 4:**
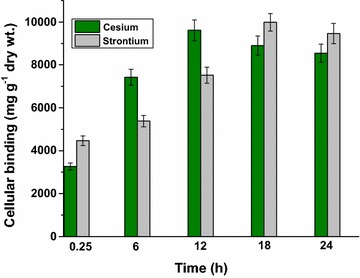
Cesium and strontium binding. Exponential phase cells equivalent of 0.28 mg dry wt. mL^−1^ (for Cs^+^) or 0.25 mg dry wt. mL^−1^ (for Sr^2+^) were exposed to 75 mM of CsCl or SrCl_2_ in 50 mL of experimental medium (LPM) at pH 7.5 and the soluble Cs^+^ or Sr^2+^ concentrations were assayed after specified time intervals. Representative time curve describing the binding capacity of the cells for Cs^+^ and Sr^2+^ has been shown

Cs^+^ and Sr^2+^ binding by KMSZP6 cells was examined following 24 h of exposure to 75 mM of CsCl or SrCl_2_ at pH 7.5. This concentration of the metals was challenging but not inhibitory for the growth of the cells. Both cesium and strontium were found to be stable and did not precipitate under the experimental conditions. Measurement of soluble Cs or Sr showed that the cells accumulated the metals gradually as a function of time when exposed to non-inhibitory concentrations of these metals (Fig. 4). Cesium binding increased from 3265 mg g^−1^ dry wt. within 15 min of exposure to 9612 mg g^−1^ dry wt. by 12 h beyond which it attained saturation. The cells sequestered 4469 mg Sr g^−1^ dry wt. within 15 min of exposure to 75 mM Sr and saturated by 18 h loading up to 9989 mg Sr g^−1^ dry wt. KMSZP6 cells demonstrated greater binding efficiency for Sr^2+^ as compared to Cs^+^ when exposed to equivalent concentration of either of the metals i.e. 75 mM. The heat killed cells showed no more than 4770 mg g^−1^ dry wt. of Cs binding and 5783 mg g^−1^ dry wt. of Sr binding within 15 min of exposure which remained almost constant over 24 h under similar experimental conditions.

Components of Cs or Sr K X-rays were detected by EDXRF analyses of the cells exposed to 75 mM Cs or Sr for 24 h when compared to the control cells, not exposed to Cs or Sr, clearly confirming the accumulation of these metals by the test organism (Additional file 4: Fig. S4). While the spectrum of cesium loaded cells revealed the presence of Cs K_α_ and K_β_ X-rays at 30.6 keV and 34.9 keV respectively, Sr K_α_ and K_β_ X-rays were observed at 14.2 and 15.8 keV respectively in the spectrum of strontium loaded biomass.

### Field emission-scanning electron microscopy (FE-SEM) and EDS analyses

**Fig. 5:**
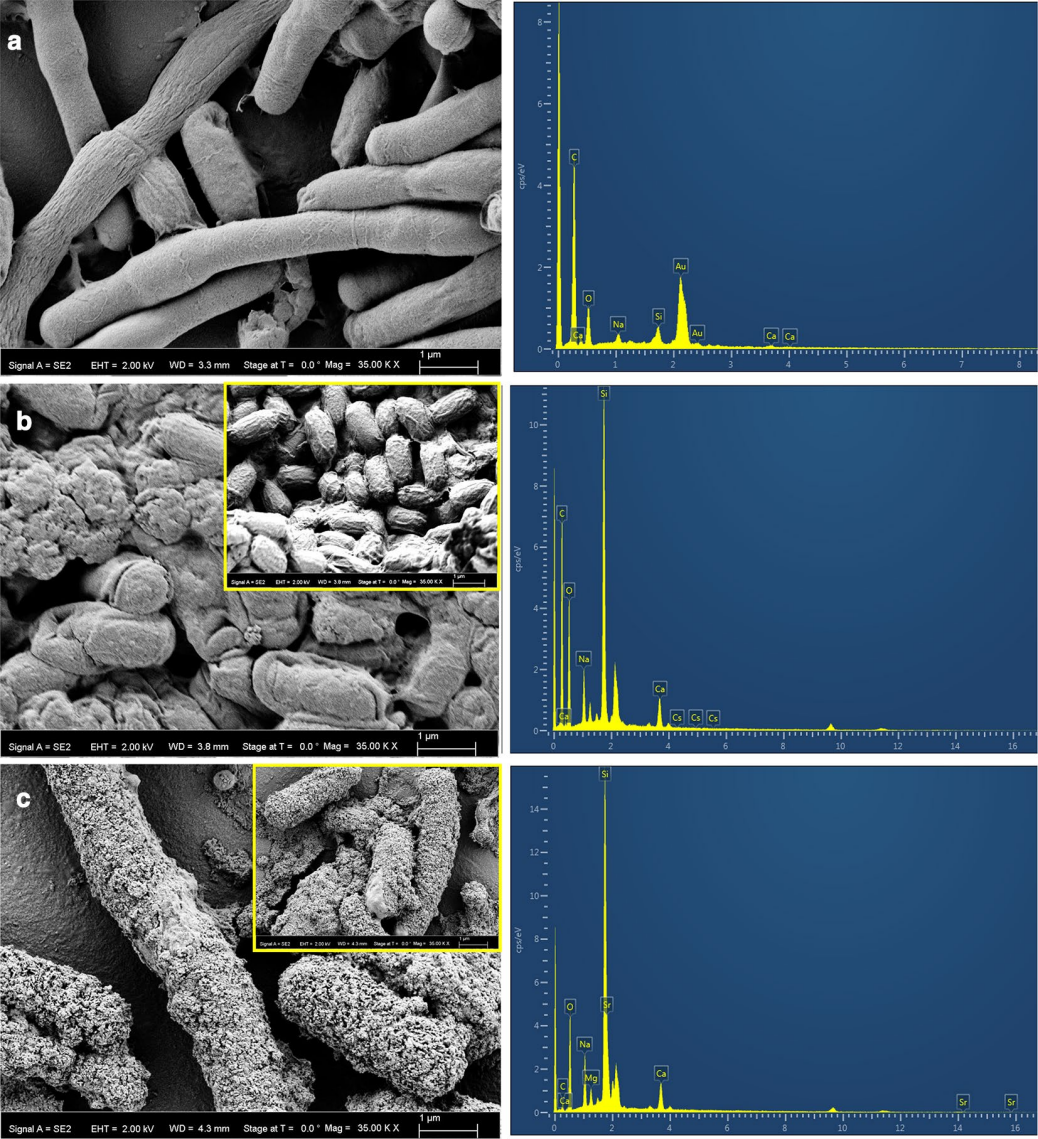
FE-SEM examination of *Arthrobacter* KMSZP6 cells exposed to cesium or strontium. Cells were incubated under **a** control conditions or **b** challenged with 75 mM of CsCl for 24 h or **c** challenged with 75 mM of SrCl_2_ for 24 h. The resulting samples were examined by field emission-scanning electron microscopy (FE-SEM) provided with energy dispersive X-ray (EDX) spectroscopy. Insets in Fig. 5b and c show another field of observation of Cs or Sr exposed cells

High resolution FE-SEM was employed to visualize the morphological changes in KMSZP6 cells following the exposure to 75 mM of cesium or strontium for 24 h (Fig. 5). Control, metal unchallenged samples exhibited elongated rod cells with smooth surfaces (Fig. 5a). The EDS analysis of these cells showed oxygen (O), calcium (Ca), sodium (Na) and carbon (C) (Fig. 5a). Cells challenged with Cs^+^ for 24 h exhibited extremely rough and contorted surfaces (Fig. 5b and inset). A striking morphological feature displayed by Cs^+^ treated cells was the predominance of shorter rods and coccoid cells as compared to the elongated rods, visualized in control cells, not exposed to Cs^+^. The genus *Arthrobacter* is characterized by pleomorphism, forming either coccoid or rod-shaped cells. Morphogenesis of *Arthrobacter* from rod to coccus has been implicated in the ability of the bacterium to survive stress (Ensign 1970). The formation of the shorter rods or coccoid cells on exposure to cesium may have enabled *Arthrobacter* to sustain the metal toxicity, ensuring its survival under unusually high concentrations of Cs^+^. SEM observations of Sr^2+^ treated cells showed a presence of both short and elongated rods. Sr^2+^ deposits were clearly visible on the surfaces of these cells when compared to cells not treated with Sr^2+^ (Fig. 5c and inset). EDS analyses of the metal treated cells displaying Cs and Sr peaks confirmed the association of these metals with KMSZP6 cells (Fig. 5b, c). The gold (Au) and silica (Si) peaks in the spectra in Fig. 5 originated from gold sputtering of the specimen and the silica grid used for support of the specimen respectively. The EDX spectra of Cs^+^ or Sr^2+^ treated cells did not exhibit any phosphorus peak excluding the likely possibility of precipitation of the metals as phosphates. These findings suggest that the association of cesium or strontium with *Arthrobacter* sp. KMSZP6 was primarily through adsorption or accumulation rather than precipitation.

FE-SEM analysis of the cells incubated with 400 mM NaCl for 24 h did not reveal any morphological variations (Additional file 5: Fig. S5) and were comparable to control cells, untreated with NaCl or any other metal (Fig. 5a). This observation is consistent with spot (Fig. 2a) and flow cytometric assays (data not included).

## Discussion

We evaluated the tolerance of a naturally occurring, uranium-resistant *Arthrobacter* strain for cesium and strontium. Metal contaminated sites are known to be colonized by persistent microbial strains which overcame metal toxicity by developing efficient molecular and cellular adaptation mechanisms allowing them to survive under stressful conditions. Such strains exhibit elevated metal tolerance.

Cesium and strontium, which are produced by the fission of uranium or plutonium in relatively high yields, are among the most hazardous radiotoxic contaminants for the environment (Ngwenya and Chirwa 2010). Highly metal resistant *Cupriavidus metallidurans* str. CH34 isolated from the sludge of a zinc processing factory showed a MIC of 125 mM for CsCl and 200 mM for SrCl_2_ (Monsieurs et al. 2011). Similarly, Cs^+^ resistant *Serratia* sp. isolated from a nuclear fuel storage pond exhibited a MIC of 400 mM for CsCl (Dekker et al. 2014). However, these studies neither evaluated Cs or Sr binding capacities of such highly tolerant organisms or measured the concentration of these radionuclides prevalent in the sources of their isolation. The strain *Arthrobacter* sp. KMSZP6 displayed noteworthy tolerance to cesium and strontium exhibiting a MIC of 400 mM for Cs and Sr. While the growth and viability of the cells were completely inhibited at 400 mM of CsCl or SrCl_2_ (Figs. 2b, c; 3), equivalent concentration of NaCl (400 mM) did not show any discernible effect on growth, viability or morphology of KMSZP6 cells (Fig. 2a; Additional file 5: Fig. S5). This finding confirmed that the observed sensitivity in KMSZP6 cells (Figs. 2b, c; 3) was due to Cs or Sr toxicity and not because of osmotic stress induced by high ionic strength of the respective metal solutions (0–400 mM CsCl or SrCl_2_). The Cs or Sr loading values exhibited by KMSZP6 cells (9612 mg Cs g^−1^ dry weight of cells or 9989 mg Sr g^−1^ dry weight of cells) are much higher than those reported for other microbes (Avery et al. 1991; Avery and Tobin 1992; Tomioka et al. 1992; Dabbagh et al. 2007; Ngwenya and Chirwa 2010), plant based bioadsorbents (Chakraborty et al. 2007) or other adsorbents like zeolite or mesoporous silica (Sangvanich et al. 2010; Sato et al. 2011) until now. The sequestration of Cs and Sr by heat killed KMSZP6 cells was only 50–60 % of that observed for live, metabolically active cells emphasizing the importance of cell viability for optimal cesium and strontium binding. Microbial Cs^+^ and Sr^2+^ accumulation is known to be mediated by cellular K^+^ and Ca^2+^ transport systems (Bossemeyer et al. 1989; Avery et al. 1991; Avery and Tobin 1992; Heuck et al. 2010). However, the detailed molecular mechanisms for Cs and Sr uptake by KMSZP6 cells is currently unknown. The radiosensitivity of microbes often limits their bioremediation abilities in high radiation environments. The tolerance to ~1 kGy of gamma radiation exhibited by KMSZP6 cells is of relevance to active uptake of Cs and Sr from radioactive nuclear waste. The exposure/external concentration of Cs or Sr in all the aforesaid binding studies using different microorganisms ranged from 0.01 μM to 4 mM. Our data revealed superior Cs^+^ and Sr^2+^ binding capacities (9612 mg Cs g^−1^ dry weight of cells or 9989 mg Sr g^−1^ dry weight of cells) of KMSZP6 cells at an external concentration as high as 75 mM of CsCl or SrCl_2_. A significant enhancement of cesium (~50 fold) or strontium (~4 fold) accumulation was observed in *Saccharomyces cerevisiae* on increasing the external Cs or Sr concentration from 50 μM to 100 mM suggesting a positive correlation between external metal concentration and cellular binding capacity (Heuck et al. 2010).

To ensure sustainability of the nuclear technology, the use of eco-friendly nuclear waste isolation methods and development of cost-effective methods for in situ containment of radionuclides are gaining importance. There has been a growing interest to study the interaction of microorganisms with radionuclides which are largely created by anthropogenic nuclear reactions. Emphasis is on the environmental microbes isolated from metal/radionuclide enriched sites in the context of nuclear waste disposal (Neu et al. 2010). Previously, *Arthrobacter* sp. strain KMSZP6 had revealed extraordinary tolerance to radionuclide like U (VI) and heavy metals like Cu (II), Pb (II) and Cd (II) and showed the presence of metal detoxification determinants like P_IB_-Type ATPase and phosphatase (Kumar et al. 2012, 2013a). By expressing high tolerance and sequestration abilities for cesium and strontium and high radiation tolerance, KMSZP6 strain presents its suitability for bioremediation of nuclear waste. The current investigation provides important information on acclimation of a naturally soil-dwelling bacterium to metal enriched environment resulting in emergence of a highly metal-resistant strain, well adapted to harsh and anthropogenic environment. Future studies are directed towards screening of transporters for Sr^2+^ and Cs^+^ in this strain for gaining in depth understanding of the mechanisms that contribute to its survival in environment enriched with heavy metals and radionuclides.

## Additional files

10.1186/s13568-016-0247-3 Location of sampling site at Domiasiat. Kylleng Mining Site 1 (KMS1, highlighted with a yellow circle was chosen as sampling point for isolation of *Arthrobacter* sp. KMSZP6 strain and physiocochemical profiling (map created using ERDAS 2010 and ArcGIS 9.3 software).
10.1186/s13568-016-0247-3 Flow cytometric dot plots (FL1 vs FL3) showing percentage subpopulation of live, injured and killed *Arthrobacter* sp. KMSZP6 cells with varying concentrations of Cs^+^ and Sr^2+^.
10.1186/s13568-016-0247-3 Radiation tolerance of *A. nicotinovorans*. Exponential phase cells (OD_600 nm_ 1.0) were exposed to gamma radiation from ^60^Co source. Volumes of 10 µL from a dilution series of the irradiated cell culture were spotted on LPM agar plates and incubated under usual growth conditions. The cells tolerated upto 1 kGy ^60^Co-gamma rays without loss of survival.
10.1186/s13568-016-0247-3 EDXRF spectra of control, unexposed *Arthrobacter* cells or cells exposed to either 75 mM Cs^+^ to achieve a loading of 8544 mg g^−1^ dry wt in 24 h or 75 mM Sr^2+^ to achieve a loading of 9464 mg g^−1^ dry wt in 24 h. The peaks corresponding to Cs K_α_ and K_β_ X-rays were seen at 30.6 keV and 34.9 keV respectively while those of Sr K_α_ and K_β_ X-rays were observed at 14.2 and 15.8 keV respectively.
10.1186/s13568-016-0247-3 FE-SEM examination of *A. nicotinovorans* cells exposed to NaCl. Exponential phase cells (equivalent to OD_600 nm_ 1) were exposed to 400 mM of NaCl for 24 h in LPM and were observed with Field Emission-Scanning Electron microscopy (FE-SEM).

## Abbreviations

LPM: low phosphate medium
MIC: minimum inhibitory concentration
EDXRF: energy dispersive X-ray fluorescence
FE-SEM: field emission-scanning electron microscopy
EDX: energy dispersive X-ray
