# Supplementary material for: Cesium and strontium tolerant Arthrobacter sp. strain KMSZP6 isolated from a pristine uranium ore deposit
Source: AMB Express. 2016 Sep 13;6(1):69. doi: 10.1186/s13568-016-0247-3 (PMC5020004; doi:10.1186/s13568-016-0247-3)
Supplement: Supplementary file 1 — 10.1186/s13568-016-0247-3 Location of sampling site at Domiasiat. Kylleng Mining Site 1 (KMS1, highlighted with a yellow circle was chosen as sampling point for isolation of Arthrobacter sp. KMSZP6 strain and physiocochemical profiling (map created using ERDAS 2010 and ArcGIS 9.3 software). [file 13568_2016_247_MOESM1_ESM.pptx]

## Slide 1
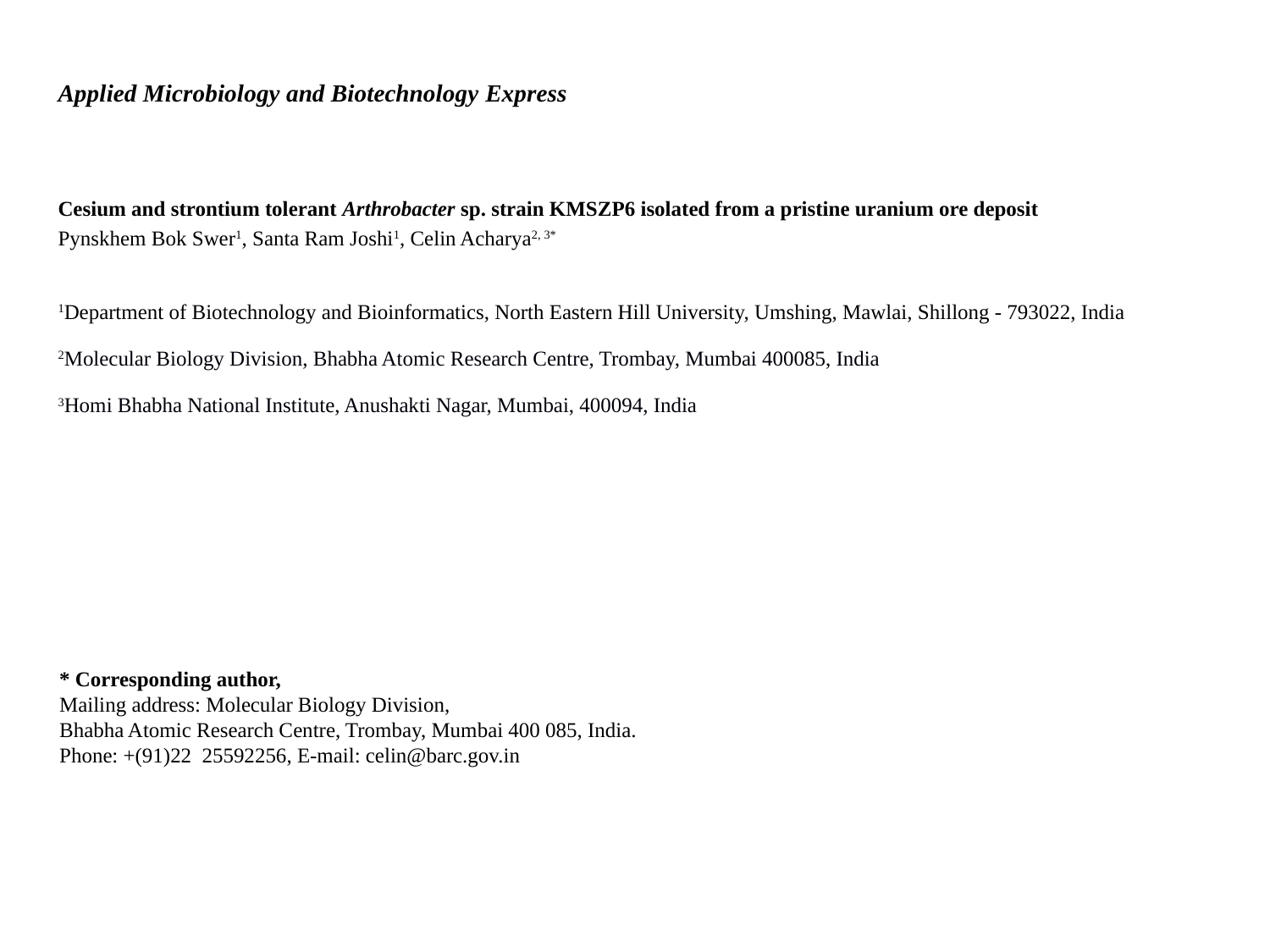

Applied Microbiology and Biotechnology Express
Cesium and strontium tolerant Arthrobacter sp. strain KMSZP6 isolated from a pristine uranium ore deposit
Pynskhem Bok Swer1, Santa Ram Joshi1, Celin Acharya2, 3*
1Department of Biotechnology and Bioinformatics, North Eastern Hill University, Umshing, Mawlai, Shillong - 793022, India
2Molecular Biology Division, Bhabha Atomic Research Centre, Trombay, Mumbai 400085, India
3Homi Bhabha National Institute, Anushakti Nagar, Mumbai, 400094, India
* Corresponding author,
Mailing address: Molecular Biology Division,
Bhabha Atomic Research Centre, Trombay, Mumbai 400 085, India.
Phone: +(91)22 25592256, E-mail: celin@barc.gov.in

## Slide 2
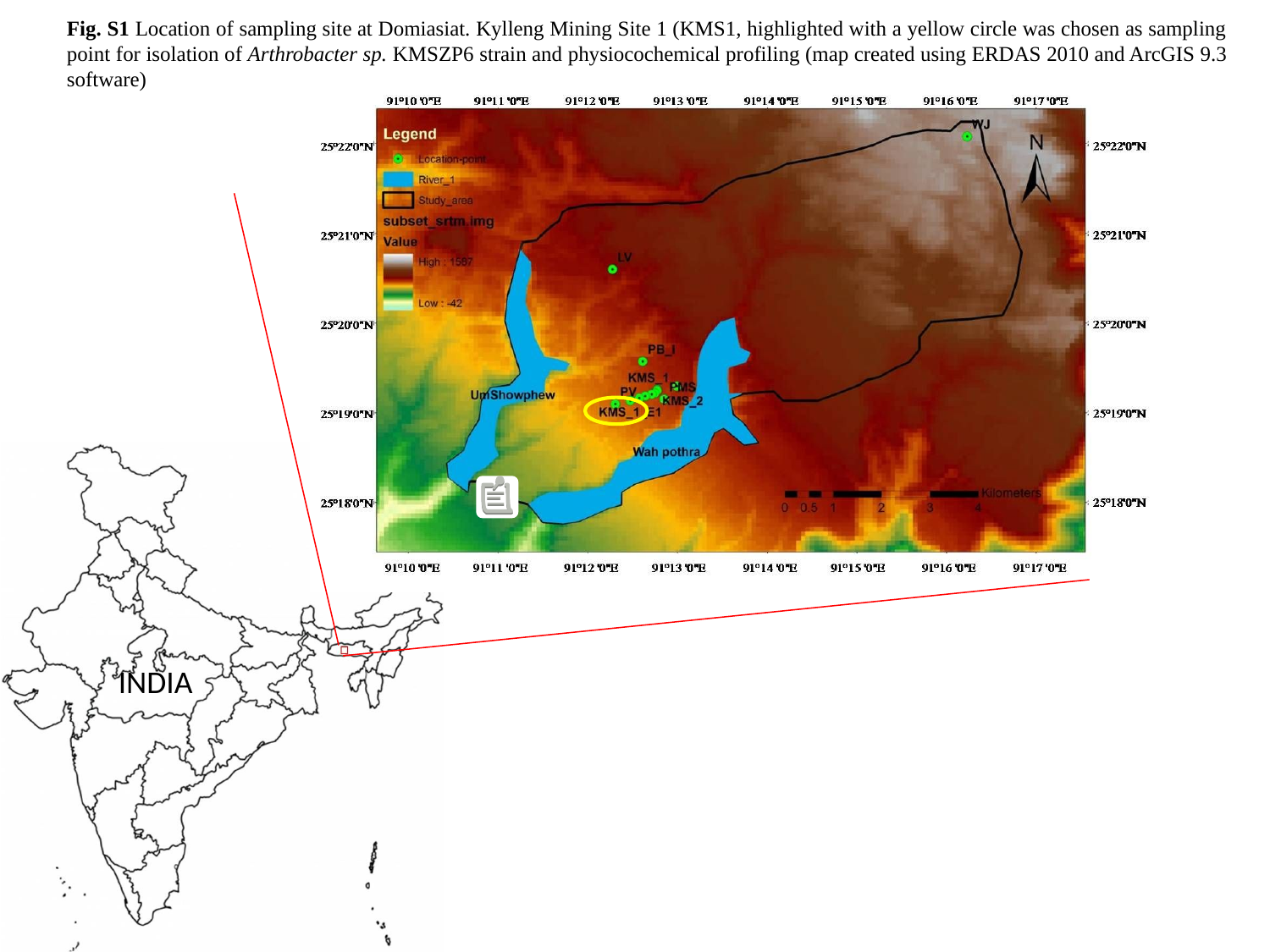

Fig. S1 Location of sampling site at Domiasiat. Kylleng Mining Site 1 (KMS1, highlighted with a yellow circle was chosen as sampling point for isolation of Arthrobacter sp. KMSZP6 strain and physiocochemical profiling (map created using ERDAS 2010 and ArcGIS 9.3 software)
INDIA
