# Supplementary material for: Cesium and strontium tolerant Arthrobacter sp. strain KMSZP6 isolated from a pristine uranium ore deposit
Source: AMB Express. 2016 Sep 13;6(1):69. doi: 10.1186/s13568-016-0247-3 (PMC5020004; doi:10.1186/s13568-016-0247-3)
Supplement: Supplementary file 4 — 10.1186/s13568-016-0247-3 EDXRF spectra of control, unexposed Arthrobacter cells or cells exposed to either 75 mM Cs+ to achieve a loading of 8544 mg g−1 dry wt in 24 h or 75 mM Sr2+ to achieve a loading of 9464 mg g−1 dry wt in 24 h. The peaks corresponding to Cs Kα and Kβ X-rays were seen at 30.6 keV and 34.9 keV respectively while those of Sr Kα and Kβ X-rays were observed at 14.2 and 15.8 keV respectively. [file 13568_2016_247_MOESM4_ESM.pptx]

## Slide 1
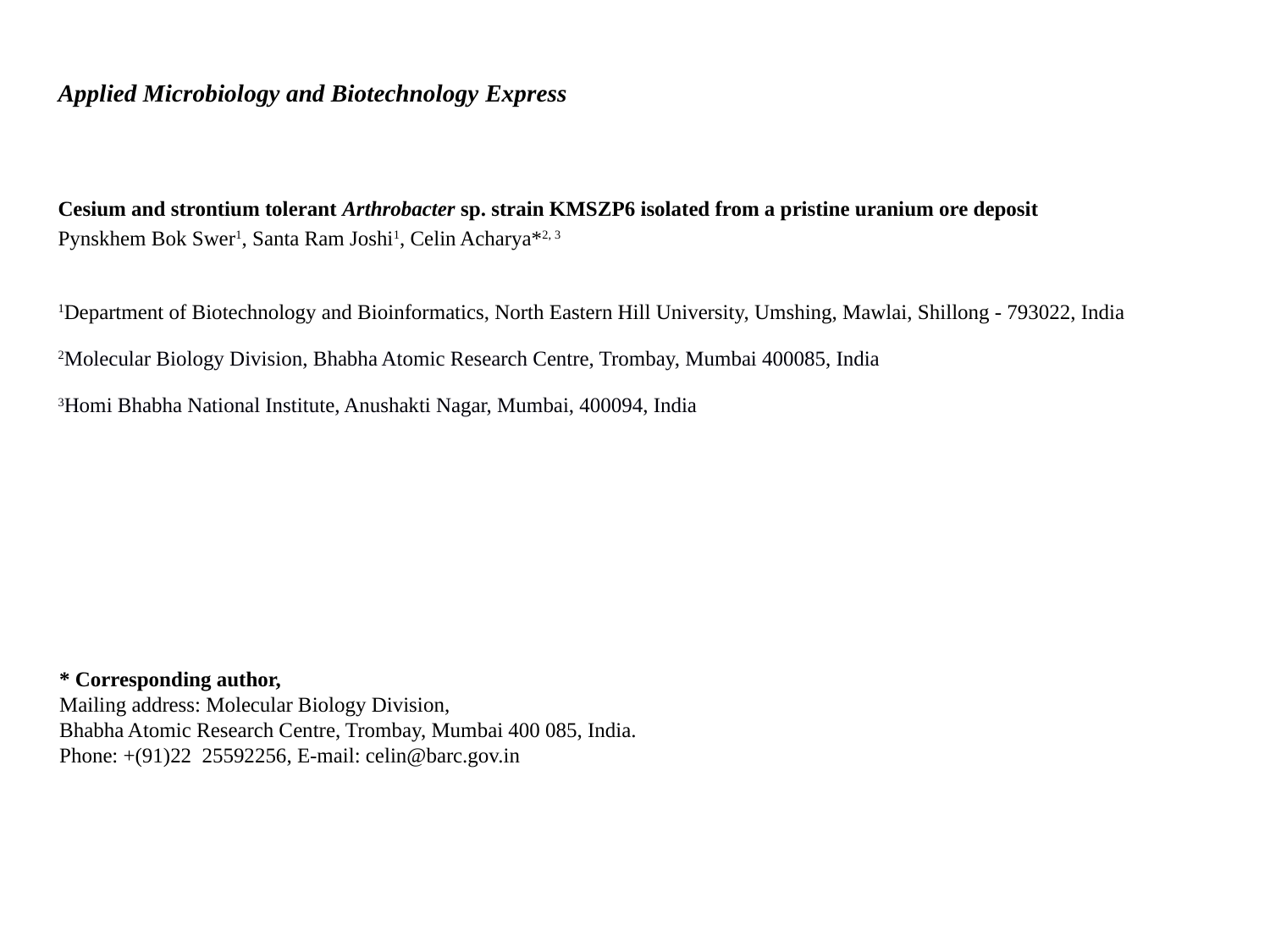

Applied Microbiology and Biotechnology Express
Cesium and strontium tolerant Arthrobacter sp. strain KMSZP6 isolated from a pristine uranium ore deposit
Pynskhem Bok Swer1, Santa Ram Joshi1, Celin Acharya*2, 3
1Department of Biotechnology and Bioinformatics, North Eastern Hill University, Umshing, Mawlai, Shillong - 793022, India
2Molecular Biology Division, Bhabha Atomic Research Centre, Trombay, Mumbai 400085, India
3Homi Bhabha National Institute, Anushakti Nagar, Mumbai, 400094, India
* Corresponding author,
Mailing address: Molecular Biology Division,
Bhabha Atomic Research Centre, Trombay, Mumbai 400 085, India.
Phone: +(91)22 25592256, E-mail: celin@barc.gov.in

## Slide 2
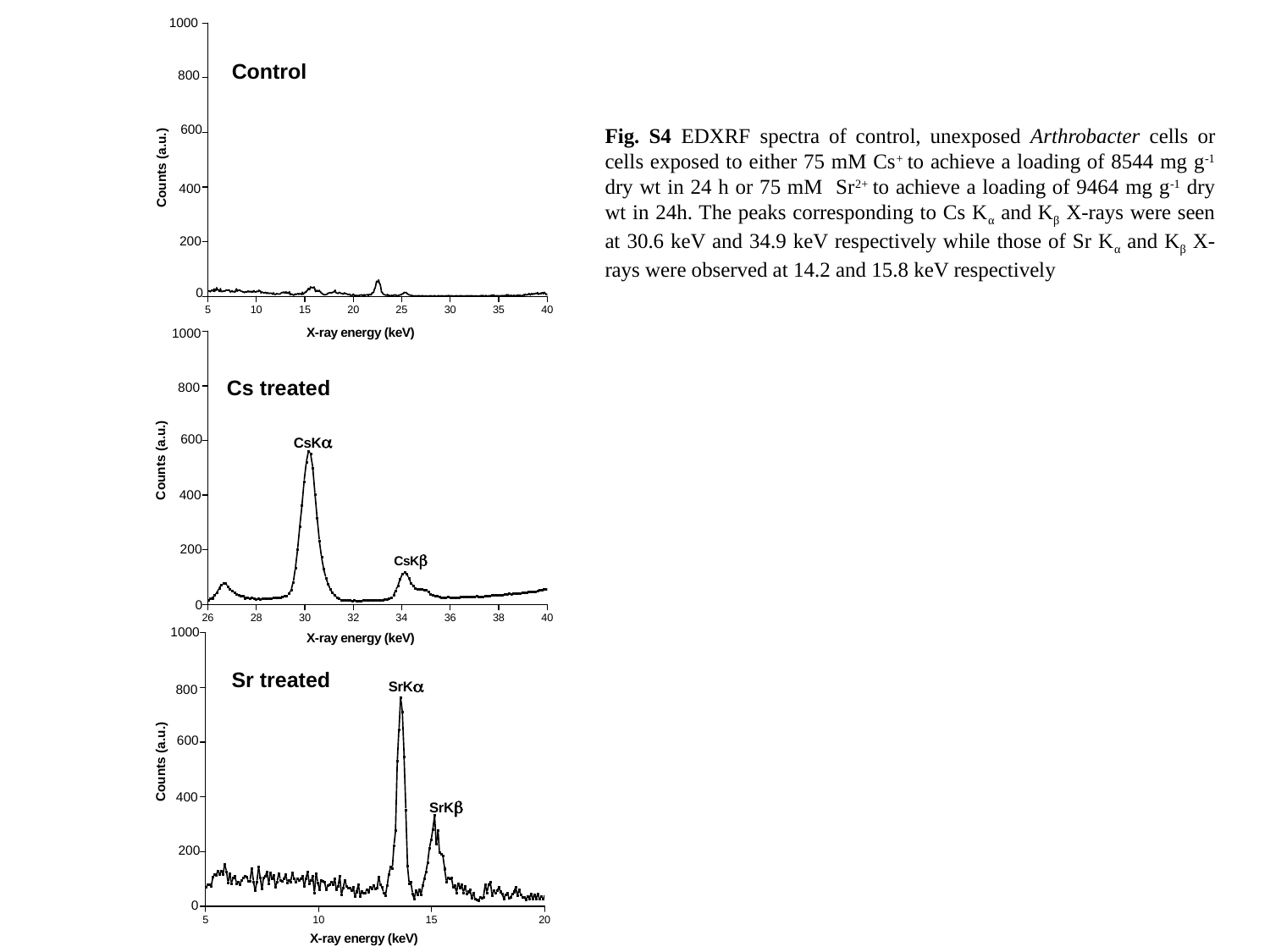

Control
Cs treated
Sr treated
Fig. S4 EDXRF spectra of control, unexposed Arthrobacter cells or cells exposed to either 75 mM Cs+ to achieve a loading of 8544 mg g-1 dry wt in 24 h or 75 mM Sr2+ to achieve a loading of 9464 mg g-1 dry wt in 24h. The peaks corresponding to Cs Kα and Kβ X-rays were seen at 30.6 keV and 34.9 keV respectively while those of Sr Kα and Kβ X-rays were observed at 14.2 and 15.8 keV respectively
